# Residential electric vehicle charging datasets from apartment buildings

**DOI:** 10.1016/j.dib.2021.107105

**Published:** 2021-04-28

**Authors:** Åse Lekang Sørensen, Karen Byskov Lindberg, Igor Sartori, Inger Andresen

**Affiliations:** a SINTEF, Department of Architectural Engineering, P.O. Box 124 Blindern, 0314 Oslo, Norway; b Norwegian University of Science and Technology (NTNU), Department of Architecture and Technology, 7491 Trondheim, Norway

**Keywords:** Electric vehicle (EV) charging, Residential electricity demand, Load profiles, End-user flexibility, Energy management

## Abstract

This data article refers to the paper "Analysis of residential EV energy flexibility potential based on real-world charging reports and smart meter data" [1]. The reported datasets deal with residential electric vehicle (EV) charging in apartment buildings. Several datasets are provided, with different levels of detail, aiming to serve various needs. The paper provides real-world EV charging reports describing 6,878 charging sessions registered by 97 user IDs, from December 2018 to January 2020. The charging reports include identifiers, plug-in time, plug-out time and charged energy for the sessions. Synthetic charging loads are provided with hourly resolution, assuming charging power 3.6 kW or 7.2 kW and immediate charging after plug-in. The non-charging idle time reflects the flexibility potential for the charging session, with synthetic idle capacity as the energy which could potentially have been charged during the idle times. Synthetic hourly charging loads and idle capacity are provided both for individual users, and aggregated for users with private or shared charge points. For a main garage with 33% of the charging sessions, smart meter data and synthetic charging loads are available, with aggregated values each hour. Finally, local hourly traffic density in 5 nearby traffic locations is provided, for further work related to the correlation with plug-in/plug-out times. Researchers, energy analysts, charge point operators, building owners and policy makers can benefit from the datasets and analyses, serving to increase the knowledge of residential EV charging. The data provides valuable insight into residential charging, useful for e.g. forecasting energy loads and flexibility, planning and modelling activities.

## Specifications Table

**Table:** 

Subject	Renewable Energy, Sustainability and the Environment
Specific subject area	Residential electric vehicle (EV) charging habits and energy loads
Type of data	CSV files Table Figure Map
How data were acquired	Obtained data, e.g. EV charging reports and Advanced Metering System (AMS) measurements, were processed using the statistical computing environment R [2]. Synthetic hourly charging loads and idle capacity were created, based on information in the charging reports and assumptions.
Data format	Raw Analysed Filtered
Parameters for data collection	Data from December 2018 to January 2020: • EV charging reports with individual charging sessions, listing identifiers, plug-in time, plug-out time and charged energy. • Hourly electricity data from AMS meters in one of the garages. • Local hourly traffic density in 5 nearby traffic locations.
Description of data collection	EV charging reports from charge point operator and hourly electricity data from grid company, both available with consent from the housing cooperative. Local hourly traffic data is downloaded from [3].
Data source location	Institution: Risvollan Housing Cooperative City/Town/Region: Trondheim Country: Norway Latitude and longitude for collected data: lat 63.395254, long 10.426319
Data accessibility	Repository name: Mendeley Data [4] Data identification number: 10.17632/jbks2rcwyj.1 Direct URL to data: http://dx.doi.org/10.17632/jbks2rcwyj.1
Related research article	Å.L. Sørensen, K.B. Lindberg, I. Sartori, I. Andresen, Analysis of residential EV energy flexibility potential based on real-world charging reports and smart meter data**,**https://doi.org/10.1016/j.enbuild.2021.110923[1].

## Value of the Data

• The datasets describe residential EV charging in apartment buildings. There is a lack of real-world data found in the literature, even though energy needs and flexibility potential are recognized.
• Researchers, energy analysts, charge point operators, building owners and policy makers can benefit from the datasets and analyses, serving to increase the knowledge of residential EV charging.
• The data provides valuable insight into residential charging, useful for e.g. forecasting energy loads and flexibility, planning and modelling activities.
• Several datasets are provided, with different levels of detail, aiming to serve various needs.
• Local traffic data is provided for further analysis, where correlation with plug-in/plug-out times can be part of new models for EV charging loads and flexibility.

## 1 Data Description

Data have been collected from a large housing cooperative in Norway, with 1,113 apartments and 2,321 residents. A new infrastructure for EV charging was installed from December 2018. From December 2018 to January 2020, charging sessions were registered by 97 user IDs; 82 of these IDs appeared to be still active at the end of the period. In the data provided with this article, Central European Time (CET) zone is used, which is GMT +1. Daylight saving time (DST) applies.

### 1.1 Dataset 1: EV charging reports

The CSV file “Dataset 1” describes 6,878 individual charging sessions, registered by 97 user IDs from December 2018 to January 2020. The charging reports include plug-in time, plug-out time and charged energy per charging session. Each charging session is connected to a user ID, charger ID and address. The charger IDs are either private or shared, since the charge points (CPs) are either located on the residents private parking spaces, or on shared parking areas available for all residents registered as users. Table 1 shows the parameters available for each of the charging sessions.

**Table 1:** Description Dataset 1: EV charging reports, describing each individual EV charging session.

session_ID	Unique ID for EV charging session (N=6878)
Garage_ID	ID for garage address (N = 24)
User_ID	ID for user (N=97)
User_type	CP ownership: Private or shared CPs
Shared_ID	When shared CPs used: ID for shared CP (N=12)
Start_plugin	Plug-in date and time (format 21.12.2018 10:20)
Start_plugin_hour	Clock hour for plug-in (from 00 to 23)
End_plugout	Plug-out date and time (format 21.12.2018 10:20)
End_charging_hour	Clock hour for plug-out (from 00 to 23)
El_kWh	Charged energy (kWh)
Duration_hours	Duration of the EV connection time, per charging session (decimal hours)
month_start	Plug-in month (January-December)
weekdays_start	Plug-in weekday (Monday-Sunday)
Plugin_ category	Category for plug-in time during the day. Each category lasts three hours (early/late night, morning, afternoon, evening)
Duration_category	Category for plug-in duration (<3h, 3-6h, 6-9h, 9-12h, 12-15h, 15-18h, >18h)

### 1.2 Dataset 2: Hourly EV charging loads and idle capacity, for all sessions and users individually

The CSV file “Dataset 2” describes EV charging loads and non-charging idle capacity for each user and all EV charging sessions individually. The synthetic hourly charging loads and idle capacity are created as described in [1]. Charging power 3.6 kW or 7.2 kW is assumed, with immediate charging after plug-in. The non-charging idle time reflects the flexibility potential for the charging session. Synthetic idle capacity is the energy load which could potentially have been charged during the idle times. The time period is from December 2018 to January 2020, and includes all active hours for each user (not a complete hourly time series per user, but hours with charging loads or idle capacity). Table 2 shows the parameters available.

**Table 2:** Description Dataset 2: Hourly EV charging loads and idle capacity, for all users individually.

date_from	Starting time (format 22.01.2019 19:00)
date_to	Ending time (format 22.01.2019 20:00)
User_ID	ID for user (N=97)
session_ID	Unique ID for EV charging session (N=6878)
Synthetic_3_6kW	Synthetic hourly energy load (kWh/h) assuming 3.6 kW charging power (ref. [1]), for users individually
Synthetic_7_2kW	Synthetic hourly energy load (kWh/h) assuming 7.2 kW charging power (ref. [1]), for users individually
Flex_3_6kW	Synthetic hourly idle capacity (kWh/h) assuming 3.6 kW charging power, for users individually
Flex_7_2kW	Synthetic hourly idle capacity (kWh/h) assuming 7.2 kW charging power, for users individually

### 1.3 Dataset 3: Hourly EV charging loads and idle capacity, aggregated for private or shared CPs

The CSV files “Dataset 3a” and “Dataset 3b” describe EV charging loads and idle capacity, aggregated for users with private (3a) or shared (3b) CPs. Charging power 3.6 kW or 7.2 kW is assumed, with immediate charging after plug-in. The time period is from December 2018 to January 2020, with a complete hourly time series. Table 3 shows the parameters available.

**Table 3:** Description Dataset 3a and 3b: Hourly EV charging loads and idle capacity, aggregated for users with private (3a) or shared (3b) CPs.

date_from	Starting time (format 22.01.2019 19:00)
daily_hour	Clock hour (from 00 to 23)
weekday	Weekday (Monday-Sunday)
month	Month (January-December)
Synthetic_3_6kW	Synthetic hourly energy load (kWh/h) assuming 3.6 kW charging power, aggregated for users with private (2a) or shared (2b) CPs
Synthetic_7_2kW	Synthetic hourly energy load (kWh/h) assuming 7.2 kW charging power, aggregated for users with private (2a) or shared (2b) CPs
Flex_3_6kW	Synthetic hourly idle capacity (kWh/h) assuming 3.6 kW charging power, aggregated for users with private (2a) or shared (2b) CPs
Flex_7_2kW	Synthetic hourly idle capacity (kWh/h) assuming 7.2 kW charging power, aggregated for users with private (2a) or shared (2b) CPs
2a: n_ private	Number of registered User IDs using private CPs (increasing, 1 to 58)
2b: n_ shared	Number of registered User IDs using shared CPs (increasing, 1 to 24)

### 1.4 Dataset 4: Average EV charging loads per user, for each daily hour during weekdays/Saturdays/Sundays

Dataset 4 in Table 4 shows average EV charging loads per user, for each daily hour during weekdays, Saturdays, and Sundays. Charging power 7.2 kW is assumed, with immediate charging after plug-in. In the table, charging loads for users with private and shared CPs are shown separately. The daily charging load profiles are based on the period with 30 to 82 users, from June 2019 to January 2020, with the number of users with private CPs increasing from 18 to 58, and users with shared CPs increasing from 12 to 24. The subset of the period is chosen, to get a more representative overview of expected power per user for aggregated loads.

**Table 4:** Average EV charging loads per user, for each daily hour during weekdays, Saturdays, and Sundays.

CP ownership	Private CPs located on residents' private parking spaces			Shared CPs available for all residents registered as users		
Daily hour	Weekdays (kWh/h/user)	Saturdays (kWh/h/user)	Sundays (kWh/h/user)	Weekdays (kWh/h/user)	Saturdays (kWh/h/user)	Sundays (kWh/h/user)
00 - 01	0.28	0.33	0.18	0.21	0.21	0.20
01 - 02	0.17	0.18	0.14	0.16	0.15	0.18
02 - 03	0.09	0.11	0.13	0.12	0.11	0.13
03 - 04	0.06	0.09	0.10	0.08	0.09	0.10
04 - 05	0.03	0.08	0.06	0.04	0.06	0.07
05 - 06	0.02	0.04	0.03	0.02	0.04	0.06
06 - 07	0.01	0.02	0.04	0.01	0.01	0.05
07 - 08	0.02	0.01	0.03	0.01	0.00	0.04
08 - 09	0.05	0.05	0.04	0.05	0.01	0.05
09 - 10	0.06	0.07	0.04	0.07	0.04	0.05
10 - 11	0.06	0.05	0.04	0.09	0.07	0.06
11 - 12	0.06	0.08	0.03	0.11	0.07	0.11
12 - 13	0.09	0.15	0.07	0.13	0.07	0.14
13 - 14	0.10	0.19	0.17	0.13	0.12	0.17
14 - 15	0.15	0.22	0.33	0.16	0.11	0.17
15 - 16	0.27	0.35	0.46	0.18	0.13	0.23
16 - 17	0.54	0.41	0.48	0.27	0.12	0.22
17 - 18	0.60	0.49	0.52	0.24	0.17	0.25
18 - 19	0.54	0.45	0.56	0.22	0.21	0.30
19 - 20	0.51	0.44	0.66	0.24	0.24	0.35
20 - 21	0.57	0.43	0.65	0.28	0.23	0.32
21 - 22	0.54	0.27	0.63	0.27	0.26	0.27
22 - 23	0.48	0.24	0.53	0.26	0.25	0.24
23 - 24	0.40	0.18	0.41	0.24	0.21	0.23
Total	5.7	4.9	6.3	3.6	3.0	4.0

### 1.5 Dataset 5: Hourly smart meter data from garage Bl2

The EVs were parked in 24 locations, whereof 22 locations have an AMS-meter measuring aggregated EV-charging at that location, with hourly resolution. This article includes AMS-measurements from a main garage, where 33% of the charging sessions took place (2,243 charging sessions). The CSV file “Dataset 5” describes hourly smart meter data from garage Bl2, with aggregated electricity use each hour. The dataset also includes synthetic hourly energy loads, aggregated for the same garage. The time period for the dataset is from January 2019 to January 2020, with a complete hourly time series. Table 5 shows the parameters available.

**Table 5:** Description Dataset 5: Hourly smart meter data from garage Bl2.

Garage_ID	ID for garage address (Bl2)
date_from	Starting time (format 22.01.2019 19:00)
date_to	Ending time (format 22.01.2019 20:00)
month	Measurement starting month
AMS_kWh	Aggregated electricity use in the garage each hour, measured by AMS meter
Synthetic_3_6kW	Synthetic hourly energy load (kWh/h) assuming 3.6 kW charging power, aggregated for users in the garage
Synthetic_7_2kW	Synthetic hourly energy load (kWh/h) assuming 7.2 kW charging power, aggregated for users in the garage
Simultaneous_if_3_6kW	Number of simultaneous charging sessions, assuming that all sessions charge with 3.6 kW charging power. NA if no charging sessions are assumed

### 1.6 Dataset 6: Local traffic density

The CSV file “Dataset 6” describes local hourly traffic density in 5 nearby traffic locations, downloaded from [3]. The data includes an hourly count of vehicles shorter than 5.6 meter, from December 2018 to January 2020. Table 6 shows the parameters available.

**Table 6:** Description Dataset 6: Local hourly traffic density.

Date_from	Starting time (format 22.01.2019 19:00)
Date_to	Ending time (format 22.01.2019 20:00)
Location 1 to 5	Number of vehicles shorter than 5.6 meter each hour, in 5 nearby traffic locations

## 2 Experimental Design, Materials and Methods

The data are analysed using the statistical computing environment R [2].

### 2.1 Dataset 1: EV charging reports

EV charging reports are received from the housing cooperative's charge point operator. Several subdivided reports are added together and organised. For each individual charging session (session_ID), plug-in time (Start_plugin), plug-out time (End_plugout) and charged energy (El_kWh) are known, as well as user ID (User_ID), CP ownership (User_type, Shared_ID) and garage location (Garage_ID). The difference between the plug-in and plug-out times of the charging sessions, provides the duration of the EV connection time (Duration_hours). Clock- and calendar data are added to the dataset (Start_plugin_hour, End_charging_hour, month_start, weekdays_start), as well as categorical values for plug-in time and plug-in duration (Plugin_ category, Duration_category).

The original EV charging reports have 7,245 charging sessions. The main steps of data cleaning include removing unrealistic charging sessions (1 CP with 29 charging sessions removed) and charging sessions with no energy charged (338 charging sessions removed). If the plug-out time is too early, compared to energy charged and maximum 11 kW charging power available, the plug-out time is removed (set to NA), since this indicates that the value is incorrect (relevant for 34 charging sessions). Further, there was quality assurance to assure correct data time zones/DST, before calendar data was added. The final dataset includes 6,878 individual charging sessions (95%).

### 2.2 Dataset 2: Hourly EV charging loads and idle capacity, for all sessions and users individually

Dataset 2 includes hourly EV charging loads and idle capacity, for all sessions and users individually. The dataset includes all active hours for each user, which are all hours the users are connected to the CP. The synthetic hourly charging loads and idle capacity are created as described in [1]. Since the actual charging time and charging power are not known, two alternative charging powers are assumed: 3.6 or 7.2 kWh/h, representing typical levels for the onboard charger capacities. The assumed charging power is the average charging power during an hour.

Synthetic hourly charging loads and idle capacity are created per charging session for all the users, assuming immediate charging after plug-in. Table 7 shows the method used to develop synthetic hourly charging loads for the charged energy (El_kWh). *P_charging_* is assumed charging power, *E_Charged_* is charged energy during the charging session (El_kWh), *E_first hour_* is energy charged during the first clock hour connected, *E_middle hours_* is energy charged during full hours charging, *E_last hour_* is energy charged during the last clock hour. The table includes an example session (Session_ID 4).

**Table 7:** Method to develop synthetic hourly charging loads.

Charged energy	Method to develop synthetic hourly charging loads	Example, Session_ID 4, assuming *P_charging_* 3.6 kWh/h
*E_first hour_*	Number of minutes after plug-in is counted. Potential energy is calculated, for a given *P_charging_*. If *E_charged_* is larger than energy potential, *E_first hour_* equals energy potential. If not, *E_first hour_* is *E_charged_*	Plug-in at 16:15: Up to 45 min charging (2.7 kWh). Since *E_charged_* is 15.56 kWh, *E_first hour_* is 2.7 kWh.

*E_middle hours_*	Remaining energy charged is calculated, as difference between *E_charged_* and *E_first hour_*. Remaining energy is divided on *P_charging_*, to get number of full hours charging with *P_charging_*.	Remaining energy: 12.86 kWh. *E_middle hours_*: 3.6 kWh/h for 3 h. Remaining energy: 2.06 kWh.

*E_last hour_*	Remaining energy will be charged.	*E_last hour_*: 2.06 kWh (34 min). Total charging time: 4 h 19 min

The difference (non-charging idle time) between the duration of the EV connection time and the assumed charging time, reflects the flexibility potential for the charging session. The idle capacity is the energy which could potentially have been charged during the non-charging idle times. Table 8 shows the method used to develop synthetic hourly idle capacity, multiplying idle time each hour with charging power. *Flex_first hour_* is idle capacity during the first clock hour with idle time, *Flex_middle hours_* is idle capacity during full hours with idle time, *Flex_last hour_* is idle capacity during the last clock hour with idle time. Also this table includes an example session (Session_ID 4).

**Table 8:** Method to develop synthetic hourly idle capacity.

Flexible energy	Method to develop synthetic hourly idle capacity	Example, Session_ID 4, assuming *P_charging_* 3.6 kWh/h
*Flex_first hour_*	Number of minutes needed to charge *E_last hour_* is calculated. If plug-out time is after needed charging time, then the charging session has idle time. *Flex_first hour_* is calculated for the available idle minutes the first hour, for a given *P_charging_*.	Connection time: 24 h 25 min. Total charging time: 4 h 19 min. Since *E_last hour_* is 2.06 kWh, *Flex_first hour_*: 1.54 kWh (26 min).

*Flex_middle hours_*	Remaining idle time is calculated, as difference between session connection time, total charging time and idle time first hour. Number of full idle hours is multiplied with *P_charging_*.	Remaining idle time: 19 h 40 min. *Flex_middle hours_*: 3.6 kWh/h for 19 h. Remaining idle time: 40 min.

*Flex_last hour_*	Remaining idle minutes is multiplied with *P_charging_*.	*Flex_last hour_*: 2.41 kWh (40 min). Total idle capacity: 72.35 kWh.

For the synthetic hourly charging loads, the synthetic charging time can become equal to or even longer than the actual connection time. If so, there is no non-charging idle time included. Also, when the plug-out time is removed in the initial data cleaning (set to NA), there is no non-charging idle time included.

### 2.3 Dataset 3: Hourly EV charging loads and idle capacity, aggregated for private or shared CPs

Dataset 3 describes EV charging loads and idle capacity, aggregated for users with private or shared CPs. First, Dataset 2 is divided on users classified as private or shared (User_type). Two hourly aggregated databases are then created by grouping the data per hour. Hours with no charging are added to the aggregated databases, to assure a full hourly timeseries for the period, from mid-December 2018 to end-January 2020.

Information about the number of registered users each day is added to the databases. The users are classified as active from the date of their first charging session (user has value NA before and 1 after first connection). In addition, some users become inactive, if they for example move or if a user using shared CPs becomes a user with private CP. Users with NA values towards the end of the measurement period are therefore classified as inactive and not included in the number of EV users. The change of classification takes place after their last charging session, from their first inactive date. However, during the last month (January 2020), only users not charging at all during the month were classified as inactive, to avoid wrong classification of users travelling etc.

### 2.4 Dataset 4: Average EV charging loads per user, for each daily hour during weekdays/Saturdays/Sundays

To create average hourly EV charging loads per user in Dataset 4, aggregated values in dataset 3 are divided on the number of users each hour. Averages for weekdays, Saturdays and Sundays are calculated for each daily hour.

**Fig. 1:**
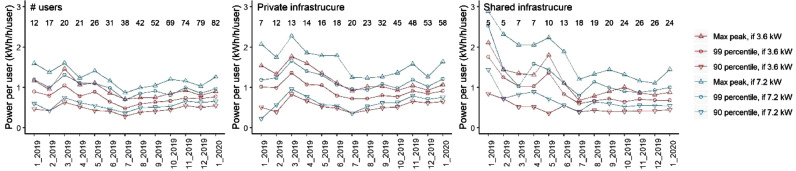
Estimated aggregated power per user, with increasing number of users, assuming charging power 3.6 kW and 7.2 kW. Left: All users, Middle: Users using private CPs, Right: Users using shared CPs.

The daily charging load profiles are based on the period with 30 to 82 users only, with the number of users with private CPs increasing from 18 to 58, and users with shared CPs increasing from 12 to 24. The subset of the period is chosen, to get a more representative overview of expected power per user for aggregated loads. Fig. 1 shows the monthly peak values per user, where the period June 2019 to January 2020 is included when calculating the average hourly EV charging loads. The figure shows how the peak power per user is reduced with increasing number of users, due to a lower coincidence factor.

### 2.5 Dataset 5: Hourly smart meter data from garage Bl2

Dataset 5 describes hourly AMS meter data for garage Bl2, measuring aggregated charging in the garage each hour. Hourly energy estimates provided by the DSO are removed from the data (8 values changed to NA), since inaccurate hourly values may influence the results. The time period for the dataset is from January 2019 to January 2020, with a complete hourly time series.

Synthetic hourly charging loads are also added to the dataset, aggregated for the garage. Finally, the dataset includes a count of the number of simultaneous charging sessions. The count is done when grouping the charging sessions each hour. For the count, it is assumed that all sessions charge with 3.6 kW charging power. The values in the column are NA if there are no counted charging sessions.

### 2.6 Dataset 6: Local traffic density

**Fig. 2:**
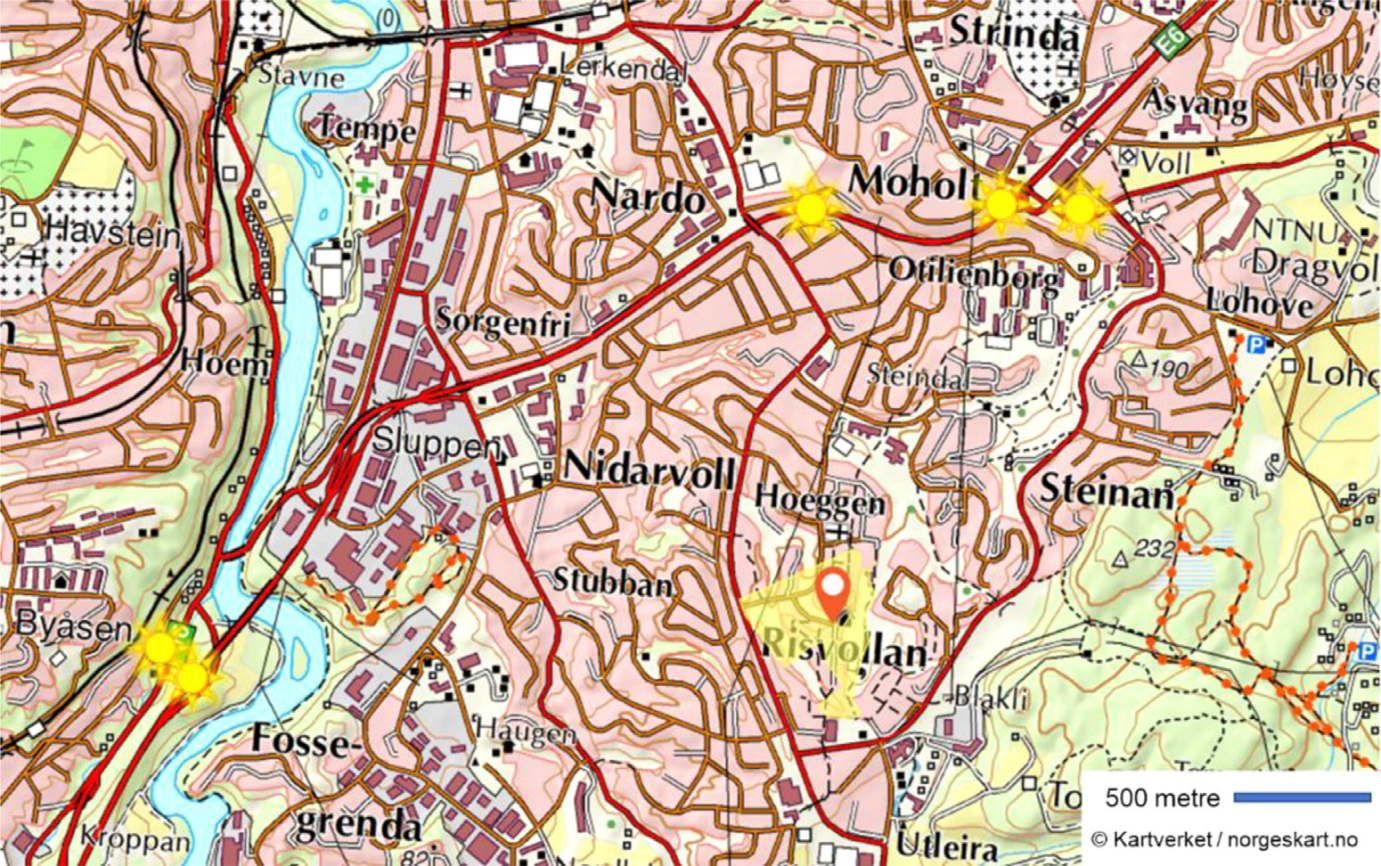
Position of the 5 locations with hourly traffic data from [2] (yellow stars) and the housing cooperative (red marker). Map: © Kartverket/norgeskart.no.

Dataset 6 describes local hourly traffic density in 5 nearby traffic locations: KROPPAN BRU, MOHOLTLIA, SELSBAKK, MOHOLT RAMPE 2, Jonsvannsveien vest for Steinanvegen. The traffic data is downloaded from [3], where traffic data is counted for vehicles with different sizes. The hourly number of small cars (less than 5.6 m) is used in the analysis, as an hourly average of the traffic measured by the five traffic stations. The geographic locations of the traffic stations and the housing cooperative are shown in the map in Fig. 2.

## Ethics Statement

Data are provided with consent from the housing cooperative and charge point operator NTE Marked. EV charging reports are anonymized.

## CRediT Author Statement

**Åse Lekang Sørensen**: Conceptualization, Methodology, Investigation, Data Curation, Writing- Original draft preparation; **Karen Byskov Lindberg**: Conceptualization, Writing - Review & Editing, Supervision; **Igor Sartori**: Conceptualization, Writing - Review & Editing, Supervision; **Inger Andresen**: Conceptualization, Writing - Review & Editing, Supervision.

## Declaration of Competing Interest

The authors declare that they have no known competing financial interests or personal relationships which have or could be perceived to have influenced the work reported in this article.

## References

[1] Sørensen Å., Lindberg K.B., Sartori I., Andresen I. (2021). Analysis of residential EV energy flexibility potential based on real-world charging reports and smart meter data. Energy Build.

[2] The R Foundation for Statistical Computing Platform, R version 3.6.2, (2019).

[3] Statens vegvesen (2020). Trafikkdata. https://www.vegvesen.no/trafikkdata.

[4] Sørensen Å.L. (2021). Data files: Residential electric vehicle charging datasets from apartment buildings.

